# Prevalence and genotype distribution of HPV and cervical pathological results in Sichuan Province, China: a three years surveys prior to mass HPV vaccination

**DOI:** 10.1186/s12985-020-01366-2

**Published:** 2020-07-10

**Authors:** Qing Luo, Ni Jiang, Qiaoyuan Wu, Jiaqiang Wang, Jialing Zhong

**Affiliations:** 1 Departments of Cancer Research Laboratory, the Affiliated Hospital of Zunyi Medical University, No.149 Dalian Road, Zunyi, 563003 Guizhou Province China; 2 Department of Laboratory Medicine, Sichuan Academy of Medical Sciences & Sichuan Provincial People’s Hospital, No. 32, West Second Section First Ring Rd., Chengdu, 610072 Sichuan China; 3 School of Medicine, University of Electronic Science and Technology of China, No. 32, West Second Section First Ring Rd, Chengdu, 610072 Sichuan China

**Keywords:** Human papillomavirus, Genotype distribution, Sichuan area, HPV vaccination

## Abstract

**Background:**

HPV persistent infection is a strong carcinogenic factor that can induce cervical cancer. Investigation of HPV epidemiology and genotype distribution is of great meaning for the development of cervical cancer prevention and control strategies.

**Methods:**

By using PCR-based hybridization gene chip assay, HPV genotype was detected from 14,185 women that came from HEC (Health Examination Center) or OGOC (Obstetrics and Gynecology Outpatient Clinics) between 2015 and 2017 in Sichuan area. The epidemiology and genotype distribution as well as the relationship between HPV infection and histology/cytology abnormalities were analyzed.

**Results:**

The positivity rate of HPV was 23.84%. The HPV-positive rate of OGOC group (37.62%) was significantly higher than that of HEC group (15.29%), *p* < 0.05. The prevalence of HPV reached peak at age 41–50 (5.86%) in HEC group, but at age 21–30 (14.74%) in OGOC group. Of all the HPV positive women, single genotype infection was the most common form in both HEC and OGOC group (62.06% in total screening population, 74.36% in HEC group and 54.01% in OGOC group). Three most prevalent HPV types were HPV-52 (5.02%), 58 (3.61%), and 16 (3.24%) in total screening population. Of all the HPV positive women, the top three types were HPV-52 (20.93%), CP8304 (15.32%), and 58 (14.42%) in HEC group, while were HPV-52 (21.14%), 16 (16.34%), and 58 (15.61%) in OGOC group. HPV 52/16/58 accounted for 41.84% of cytology and 56.52% of histological abnormalities.

**Conclusions:**

Women in Sichuan area were facing the great threat of HPV infection, especially the women aged between 21 ~ 30 or 41–50 years old. The priority HPV types were HPV 52, 58, and 16 in OGOC group, while were HPV 52, CP8304, and 58 in HEC group. HPV 52/16/58 accounted for the majority of cytology and histological abnormalities. Our analysis was found to be valuable for providing a scientific basis for the prevention and control strategies of cervical cancer in Sichuan area.

## Background

Cervical cancer is the fourth most common cancer and the fourth leading cause of cancer death for women, with an estimated 570, 000 case and 311,000 deaths in 2018 worldwide, which is a serious threat to female health, especially in developing countries [1]. As the most populous developing country, China has 98,900 new cases of cervical cancer and 30,500 cervical cancer-related deaths in 2015 [2]. Thus, there is a heavy disease burden of cervical cancer in china.

Human papillomaviruses (HPVs) are non-enveloped, double-stranded circular DNA viruses. To date, over 170 types of HPV have been identified, of which 40 infect the genital tract. Generally, HPVs could be divided into low-risk categories (e.g. HPV 6, 11, 42, 43, 44, CP8304) which were mainly found in the genital warts, and high-risk categories (e.g. HPV 16, 18, 31, 33, 35, 39, 45, 51, 52, 53, 56, 58, 59, 66 and 68),the persistent infection of which has been recognized as a strong carcinogenic factor to induce cervical cancer [3, 4]. Because of the huge difference regarding the ability to induce cervical cancer, it was worthwhile to investigate HPV DNA genotype to improve the effect of cervical cancer screening.

HPV vaccines have showed a great potential on the prevention of cervical cancer. The prophylactic injection of HPV vaccine can decline vaccine-type HPV infection and substantially lead to reductions in HPV-associated cancers [5]. However, the vaccines showed prominently type-restricted prophylactic efficacy. They merely induced immunity specific to certain HPV genotypes, and were unable to fend off other genotypes of the virus [4]. Studies also demonstrated that the distribution of HPV genotypes differed considerably both geographically and among populations. Therefore, to investigate the epidemiology and genotype distribution of HPV in a certain population was important for developing HPV vaccine strategy in this area [6].

Sichuan, a province in southwestern China, has the fourth largest population in the country, with a permanent population of 83.41 million in 2018(data from National Bureau of Statistics of China, http://www.stats.gov.cn/). Several studies aimed to investigate the epidemiology and genotype distribution of HPV in this area recent years, however, the results varied a lot [7–12].

In this study, in order to get a more accurate and reliable data, we audited large-scale retrospective data on cervical HPV testing among women that attended Health Examination Center (HEC) and obstetrics and gynecology outpatient clinics (OGOC) during 2015–2017. We also assessed the relationship between type-specific infection of HPV and the histology/cytology triage, Our study might provide a scientific basis for the prevention and control strategies of cervical cancer in Sichuan area.

## Methods

### Study population and include criteria

A total of 14,185 women who participated in HPV screening in HEC or OGOC of Sichuan People’s Hospital were included from 2015 to 2017. The participants were enrolled according to the following selection criteria: (1) was mentally and physically competent; (2) was sexually active women of any age; (3) was willing to undergo HPV testing. The age of the patients ranged from 14 to 78 years old with an average of 39.93 ± 10.38 years.

### Cervical sample collection

Samples exfoliated cervical cells were collected by using plastic cervical swabs. Each plastic swab was mixed well with 1 ml of specimen transport medium (Chaozhou Hybribio Biotechnology Limited Corporation, China) and store immediately at 4 °C, and finally sent to our clinical laboratory within 1 day for HPV analysis.

### HPV DNA extraction and genotyping

HPV DNA extraction and genotyping DNA was performed by using Alkaline Lysis Method Kits (Chaozhou Hybribio Biotechnology Limited Corporation, China) and HPV GenoArray Test kit (Chaozhou Hybribio Biotechnology Limited Corporation, China) according to the manufacturer’s protocol [13, 14] The GenoArray could identify 15 HR-HPV (high-risk HPV) types (16, 18, 31, 33, 35, 39, 45, 51, 52, 53, 56, 58, 59, 66 and 68) and 6 LR-HPV (low-risk HPV) types (6, 11, 42, 43, 44 and CP8304 in Chinese population). The final results were detected by colorimetric change on the GenoArray under direct visualization.

### Thin prep liquid-based cytology test

For cytology test, cervical slides were prepared using Liquid-PREP™ System (LGM International, USA). The slides were evaluated for cervical cytology by two academic cyto-pathologists of Sichuan Provincial People’s Hospital. Cytological classifications of disease grade were made in conformity to the Bethesda 2001 criteria, including negative of intraepithelial lesion or malignancy (NILM), atypical squamous cells of undetermined significance (ASCUS), atypical gland cell (AGC),low-grade squamous intraepithelial lesion (LSIL), high-grade squamous intra epithelial lesion (HSIL) and invasive cervical cancer (ICC).

### Histology diagnosis

For cervical biopsies, Digital Electronic Colposcope (Olympus Company, Japan) was used by specially trained physicians to perform the cervical biopsy tissue collection. The histology results were obtained by two academic Gynecology doctors of Sichuan Provincial People’s Hospital. A diagnosis was assigned to each case, as having NO Lesion, cervical intraepithelial neoplasia grade 1 (CIN 1), CIN 2, CIN 3, cervical cancer (CA).

### Statistical analysis

All statistical analyses were performed using SPSS 22.0 software (SPSS Inc., Chicago, IL, USA). Type-specific prevalence of HPV infection and their exact binomial 95% confidence intervals (CI) were calculated. Pearson’s χ^2^ test was performed to evaluate the significance of differences between designated groups. All analyses were two-sided and interpreted as being significant at *p* < 0.05.

## Results

### Prevalence of HPV infection in HEC and OGOC group

Overall, 14,185 women were included in this study. Among them, 3382 were HPV positive, account for 23.84%. Among the 14,185 women, 8751 came from HEC, and 5434 came from OGOC. The HPV-positive rate of OGOC group (37.62%) was significantly higher than that of HEC group (15.29%), *p* < 0.05 (Table 1).

**Table 1:** Prevalence of HPV infection in different age groups

Age Group (Year)	Total case						HPV positive cases					
	HEC		OGOC		Total		HEC		OGOC		Total	
	n	%^a^	n	%^a^	n	%^a^	n	%^b^	n	%^b^	n	%^b^
≤20	2	0.02	149	2.74	151	1.06	1	0.01	86	1.58	87	0.61
21–30	902	10.31	2111	38.85	3013	21.24	149	1.70	801	14.74	950	6.70
31–40	2722	31.11	1368	25.17	4090	28.83	404	4.62	462	8.50	866	6.11
41–50	3552	40.59	1201	22.10	4753	33.51	513	5.86	397	7.31	910	6.42
51–60	1262	14.42	457	8.41	1719	12.12	220	2.51	215	3.96	435	3.07
61–70	274	3.13	129	2.37	403	2.84	47	0.54	75	1.38	122	0.86
≥71	37	0.42	19	0.35	56	0.39	4	0.05	8	0.15	12	0.08
Total	8751	100.00	5434	100.00	14,185	100.00	1338	15.29	2044	37.62	3382	23.84

^a^: Percentage of each age group in HEC/OGOC /Total group

^b^: Percentage of HPV positive cases in each age group in HEC/OGOC /Total group

### Prevalence of HPV grouped by age in study population

All the participants were divided into seven groups according to their age (≤20 years, 21–30 years, 31–40 years, 41–50 years, 51–60 years, 61–70 years and ≥ 71 years). The prevalence of HPV reached a peak at 21–30 years and 41–50 years, with a positive rate of 6.70 and 6.42%, respectively. Specifically, the prevalence of HPV reached peak at 41–50 years group (5.86%) in HEC group, but at 21–30 years group (14.74%) in OGOC group (Table 1, supplementary Fig. 1).

### Prevalence of HPV single and multiple HPV genotypes infection

For all the HPV positive women, single genotype infection was the most (62.06% in total group, 74.36% in HEC group and 54.01% in OGOC group). Among the multiple infection cases, double genotype infection was the most, and the infection rate decreased significantly as the number of infected genotypes increased (Table 2). OGOC group showed a significantly higher prevalence rate of multiple genotype infection than HEC group (*p* < 0.05).

**Table 2:** Prevalence of HPV single/multiple infection in HEC and OGOC group

Prevalence of HPV infection	Total HPV^+^		HEC HPV^+^		OGOC HPV^+^	
	n	%^a^	n	%^a^	n	%^a^
Single Infection	2099	62.06	995	74.36	1104	54.01
Mutlple Infection	1283	37.94	343	25.64	940	45.99
Double Infection	903	26.70	267	19.96	636	31.12
Triple Infection	266	7.87	64	4.78	202	9.88
Quadruple Infection	76	2.25	11	0.82	65	3.18
Quintet Infection	22	0.65	0	0.00	22	1.08
Sextuple Infection	10	0.30	1	0.07	9	0.44
Septuple Infection	6	0.18	0	0.00	6	0.29
Total Infection	3382	100.00	1338	100.00	2044	100.00

^a^: Percentage of each infection form in HPV positive women of Total/HEC/OGOC group

### Prevalence of HPV genotypes in HPV-positive population

Among the 21 genotypes of HPV that can be identified by our GenoArray, there were 15 high-risk types (HPV16, 18, 31, 33, 35, 39, 45, 51, 52, 53, 56, 58, 59, 66, 68) and 6 low-risk types (6, 11, 42,43, 44, CP8304). In our study, the top three prevalent HPV types were HPV-52 (5.02%, 712/14184), 58 (3.61%, 512/14184), and 16 (3.24%, 460/14185) in total screening population, which followed by CP8304 (3.12%, 443/14185) and 53(2.89%, 410/14184). As shown in Table 3, among the HPV positive population, the genotypes of infected HPV varied in different groups. In HEC group, the top three were HPV-52 (20.93%), CP8304 (15.32%), 58 (14.42%), followed by 53(13.75%) and 16(9.42%). While in OGOC group, the top three were HPV-52 (21.14%), 16 (16.34%), 58 (15.61%), followed by CP8304 (11.63%) and 53(11.06%). The prevalence of HPV 16, 18, 6, 11 genotype was significantly higher in OGOC group than that in HEC group. Interestingly, the prevalence of HPV 53 and CP8304 was significantly higher in HEC group than that in OGOC group.

**Table 3:** Prevalence of HPV genotypes in HPV-positive population

	Total HPV+				HEC HPV+				OGOC HPV+				*p*^a^
	S%^c^ (*n* = 2478)	M%^d^ (*n* = 904)	T%^e^ (*n* = 3382)	CI (95%) (*n* = 3382)	S%^c^ (*n* = 1052)	M%^d^ (*n* = 267)	T%^e^ (*n* = 1319)	CI (95%) (*n* = 1319)	S%^c^ (*n* = 1426)	M%^d^ (*n* = 637)	T%^e^ (n = 2063)	CI (95%) (*n* = 2063)	
High Risk													
16	8.10	5.50	13.60	12.45–14.76	5.98	3.44	9.42	7.85–10.98	9.49	6.85	16.34	14.74–17.94	< 0.001^b^
18	2.28	2.69	4.97	4.24–5.70	2.24	1.79	4.04	2.98–5.09	2.30	3.28	5.58	4.58–6.57	0.044^b^
31	1.77	1.77	3.55	2.92–4.17	1.79	1.27	3.06	2.14–3.99	1.76	2.10	3.86	3.03–4.70	0.218
33	2.75	2.28	5.03	4.29–5.76	3.21	1.42	4.63	3.51–5.76	2.45	2.84	5.28	4.31–6.25	0.398
35	0.50	0.59	1.09	0.74–1.44	0.75	0.45	1.20	0.61–1.78	0.34	0.68	1.03	0.59–1.46	0.645
39	4.82	4.46	9.28	8.31–10.26	5.53	3.44	8.97	7.44–10.50	4.35	5.14	9.49	8.22–10.76	0.609
45	0.33	0.68	1.01	0.67–1.34	0.45	0.52	0.97	0.45–1.50	0.24	0.78	1.03	0.59–1.46	0.874
51	3.78	4.70	8.52	7.57–9.46	4.71	3.36	8.07	6.61–9.53	3.18	5.58	8.81	7.58–10.03	0.454
52	12.89	8.16	21.05	19.68–22.43	14.13	6.80	20.93	18.75–23.11	12.08	9.05	21.14	19.37–22.90	0.884
53	6.80	5.32	12.12	11.02–13.22	9.49	4.26	13.75	11.91–15.60	5.04	6.02	11.06	9.70–12.42	0.019^b^
56	1.21	1.86	3.08	2.49–3.66	1.12	1.27	2.39	1.57–3.21	1.27	2.25	3.52	2.72–4.32	0.063
58	9.05	6.09	15.14	13.93–16.35	9.42	5.01	14.42	12.54–16.31	8.81	6.80	15.61	14.03–17.18	0.348
59	1.12	1.27	2.40	1.88–2.91	0.90	0.97	1.87	1.14–2.59	1.27	1.47	2.74	2.03–3.45	0.105
66	1.98	2.25	4.23	3.55–4.91	2.54	1.42	3.96	2.92–5.01	1.61	2.79	4.40	3.51–5.29	0.532
68	2.40	2.72	5.12	4.37–5.86	2.77	2.32	5.08	3.91–6.26	2.15	2.98	5.14	4.18–6.09	0.944
Low Risk													
6	2.96	3.64	6.62	5.79–7.46	0.82	0.90	1.72	1.02–2.42	4.35	5.48	9.83	8.54–11.12	< 0.001^b^
11	2.40	2.87	5.26	4.51–6.02	1.05	0.67	1.72	1.02–2.42	3.28	4.26	7.58	6.44–8.73	< 0.001^b^
42	0.06	0.30	0.35	0.15–0.56	0.07	0.00	0.07	−0.07 ~ 0.22	0.05	0.49	0.54	0.22–0.86	0.055
43	0.18	0.30	0.47	0.24–0.70	0.15	0.07	0.22	−0.03-0.48	0.20	0.44	0.64	0.29–0.98	0.088
44	1.01	0.98	1.98	1.51–2.45	1.42	1.05	2.47	1.64–3.30	0.73	0.93	1.66	1.11–2.22	0.101
CP8304	6.89	6.21	13.10	11.96–14.24	10.09	5.23	15.32	13.39–17.25	4.79	6.85	11.64	10.25–13.03	*p* = 0.002^b^

^a^: HEC group versus OGOC (*p* value from Pearson’s χ^2^ test)

^b^: Statistical significance

^c^: women of single genotype infection;

^d^: women of multiple genotypes infection;

^e^: Total women

### Distribution of HPV genotypes according to cytology abnormalities

**Fig. 1:**
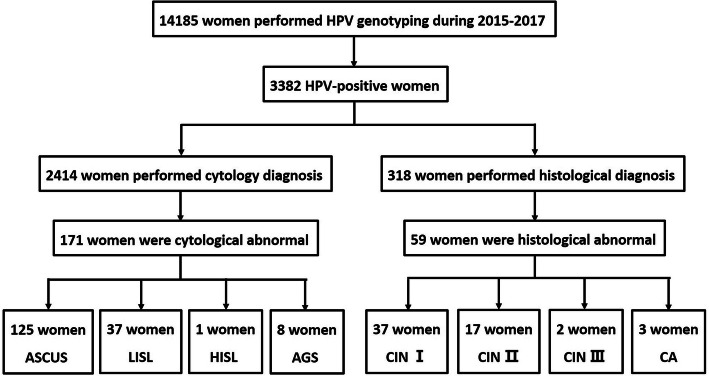
Flow chart of cytology and histological abnormalities samples

Figure 1 described the process by which we obtained our final results of cytology and histological abnormalities. Among the 3382 HPV positive women, 2414 were performed Thinprep cytologic test (TCT), and 171 women have been identified to be cytology abnormalities, accounting for 7.08%. The distribution of HPV genotypes in cytology abnormalities was showed in Table 4. Single-genotype infection was the main infection mode in ASCUS (77/125, 61.60%), LSIL (22/37, 59.46%) and HSIL (1/1, 100%) group. As for AGC group, the single-genotype infection rate was 50% (4/4). The most common infected genotype was HPV 52 (29.60%) in ASCUS group, HPV 66 (21.62%) in LSIL group, HPV 58 (100%) in both HSIL and AGC group. Prevalence of high-risk HPV is 81.41, 88.57 and 100% in ASCUS, LSIL and HSIL, respectively. The top infected genotype was HPV52 in ASCUS group, HPV 66 in LSIL group, and HPV 58 in HSIL group.

**Table 4:** Distribution of HPV genotypes according to cytology abnormalities

	ASCUS			LSIL			HSIL			AGC		
	S%^a^ (*n* = 77)	M%^b^ (*n* = 48)	T%^c^ (*n* = 125)	S%^a^ (*n* = 22)	M%^b^ (*n* = 15)	T%^c^ (*n* = 37)	S%^a^ (*n* = 1)	M%^b^ (*n* = 0)	T%^c^ (*n* = 1)	S%^a^ (*n* = 4)	M%^b^ (*n* = 4)	T%^c^ (*n* = 8)
High Risk	**81.41%**			**88.57%**			**100%**			**100%**		
16	12.80	9.60	22.40	8.11	10.81	18.92	0.00	0.00	0.00	12.50	0.00	12.50
18	0.00	2.40	2.40	5.41	8.11	13.51	0.00	0.00	0.00	0.00	12.50	12.50
31	1.60	0.00	1.60	2.70	5.41	8.11	0.00	0.00	0.00	0.00	0.00	0.00
33	4.00	5.60	9.60	2.70	5.41	8.11	0.00	0.00	0.00	12.50	12.50	25.00
35	0.80	0.00	0.80	0.00	5.41	5.41	0.00	0.00	0.00	0.00	0.00	0.00
39	2.40	4.80	7.20	2.70	16.22	18.92	0.00	0.00	0.00	0.00	12.50	12.50
45	0.00	0.80	0.80	0.00	0.00	0.00	0.00	0.00	0.00	0.00	0.00	0.00
51	0.80	6.40	7.20	2.70	0.00	2.70	0.00	0.00	0.00	0.00	37.50	37.50
52	14.40	15.20	29.60	2.70	13.51	16.22	0.00	0.00	0.00	0.00	0.00	0.00
53	1.60	6.40	8.00	5.41	8.11	13.51	0.00	0.00	0.00	0.00	0.00	0.00
56	2.40	4.80	7.20	2.70	8.11	10.81	0.00	0.00	0.00	0.00	0.00	0.00
58	8.80	12.80	21.60	8.11	10.81	18.92	100.00	0.00	100.00	25.00	25.00	50.00
59	0.80	0.80	1.60	0.00	2.70	2.70	0.00	0.00	0.00	0.00	0.00	0.00
66	0.00	6.40	6.40	13.51	8.11	21.62	0.00	0.00	0.00	0.00	0.00	0.00
68	1.60	1.60	3.20	0.00	5.41	5.41	0.00	0.00	0.00	0.00	0.00	0.00
Low Risk	**18.59%**			**11.43%**			**0.00%**			**0.00%**		
6	2.40	3.20	5.60	0.00	2.70	2.70	0.00	0.00	0.00	0.00	0.00	0.00
11	2.40	2.40	4.80	2.70	2.70	5.41	0.00	0.00	0.00	0.00	0.00	0.00
42	0.00	0.00	0.00	0.00	2.70	2.70	0.00	0.00	0.00	0.00	0.00	0.00
43	0.00	1.60	1.60	0.00	0.00	0.00	0.00	0.00	0.00	0.00	0.00	0.00
44	0.80	2.40	3.20	0.00	0.00	0.00	0.00	0.00	0.00	0.00	0.00	0.00
CP8304	4.00	10.40	14.40	0.00	13.51	13.51	0.00	0.00	0.00	0.00	0.00	0.00

^a^: women of single genotype infection;

^b^: women of multiple genotypes infection;

^c^: Total women

### Distribution of HPV genotypes according to histological abnormalities

Among the 3382 HPV positive women, 318 were performed cervical biopsy, and 59 women were identified to be histological abnormalities (accounting for 18.55%). The single-genotype infection was still the main infection mode in CIN I (21/37, 56.76%), CINII (13/17, 76.47%), CIN III (2/2, 100%) and CA (2/3, 66.67%) group. The most common genotypes are HPV 58 (35.14%), 52(27.03%), 16(21.62%) in CIN I group, HPV 16 (70.59%), 52/33(23.52, 23.52%), 51/39(11.76,11.76%) in CINIIgroup, HPV 58/16 (50, 50%) in CIN IIIand HPV16/18/51/58 (25.00, 25.00, 25.00, 25.00%) in CA group (Table 5).

**Table 5:** Distribution of HPV genotypes according to histological abnormalities

	CIN I			CIN II			CIN III			CA		
	S%^**a**^ (***n*** = 21)	M%^**b**^ (***n*** = 16)	T%^**c**^ (n = 37)	S%^**a**^ (***n*** = 13)	M%^**b**^ (***n*** = 4)	T%^**c**^ (***n*** = 17)	S%^**a**^ (***n*** = 2)	M%^**b**^ (***n*** = 0)	T%^**c**^ (***n*** = 2)	S%^**a**^ (***n*** = 2)	M%^**b**^ (***n*** = 1)	T%^**c**^ (***n*** = 3)
High Risk	**89.83%**			**96.30%**			**100%**			**100%**		
16	10.81	10.81	21.62	52.94	17.65	70.59	50.00	0.00	50.00	25.00	0.00	25.00
18	0.00	5.41	5.41	5.88	0.00	5.88	0.00	0.00	0.00	0.00	25.00	25.00
31	0.00	2.70	2.70	0.00	0.00	0.00	0.00	0.00	0.00	0.00	0.00	0.00
33	5.41	0.00	5.41	11.76	11.76	23.53	0.00	0.00	0.00	0.00	0.00	0.00
35	0.00	2.70	2.70	0.00	0.00	0.00	0.00	0.00	0.00	0.00	0.00	0.00
39	2.70	8.11	10.81	0.00	11.76	11.76	0.00	0.00	0.00	0.00	0.00	0.00
45	0.00	0.00	0.00	0.00	0.00	0.00	0.00	0.00	0.00	0.00	0.00	0.00
51	2.70	2.70	5.41	0.00	11.76	11.76	0.00	0.00	0.00	0.00	25.00	25.00
52	13.51	13.51	27.03	5.88	17.65	23.53	0.00	0.00	0.00	0.00	0.00	0.00
53	0.00	8.11	8.11	0.00	0.00	0.00	0.00	0.00	0.00	0.00	0.00	0.00
56	2.70	8.11	10.81	0.00	0.00	0.00	0.00	0.00	0.00	0.00	0.00	0.00
58	18.92	16.22	35.14	0.00	5.88	5.88	50.00	0.00	50.00	25.00	0.00	25.00
59	0.00	2.70	2.70	0.00	0.00	0.00	0.00	0.00	0.00	0.00	0.00	0.00
66	0.00	2.70	2.70	0.00	0.00	0.00	0.00	0.00	0.00	0.00	0.00	0.00
68	0.00	2.70	2.70	0.00	0.00	0.00	0.00	0.00	0.00	0.00	0.00	0.00
Low Risk	**10.17%**			**3.70%**			**0.00%**		**0.00%**			
6	0.00	0.00	0.00	0.00	5.88	5.88	0.00	0.00	0.00	0.00	0.00	0.00
11	0.00	2.70	2.70	0.00	0.00	0.00	0.00	0.00	0.00	0.00	0.00	0.00
42	0.00	0.00	0.00	0.00	0.00	0.00	0.00	0.00	0.00	0.00	0.00	0.00
43	0.00	2.70	2.70	0.00	0.00	0.00	0.00	0.00	0.00	0.00	0.00	0.00
44	0.00	2.70	2.70	0.00	0.00	0.00	0.00	0.00	0.00	0.00	0.00	0.00
CP8304	0.00	8.11	8.11	0.00	0.00	0.00	0.00	0.00	0.00	0.00	0.00	0.00

^a^: women of single genotype infection;

^b^: women of multiple genotypes infection;

^c^: Total women

### Prevalence of HPV infection change by year

**Fig. 2:**
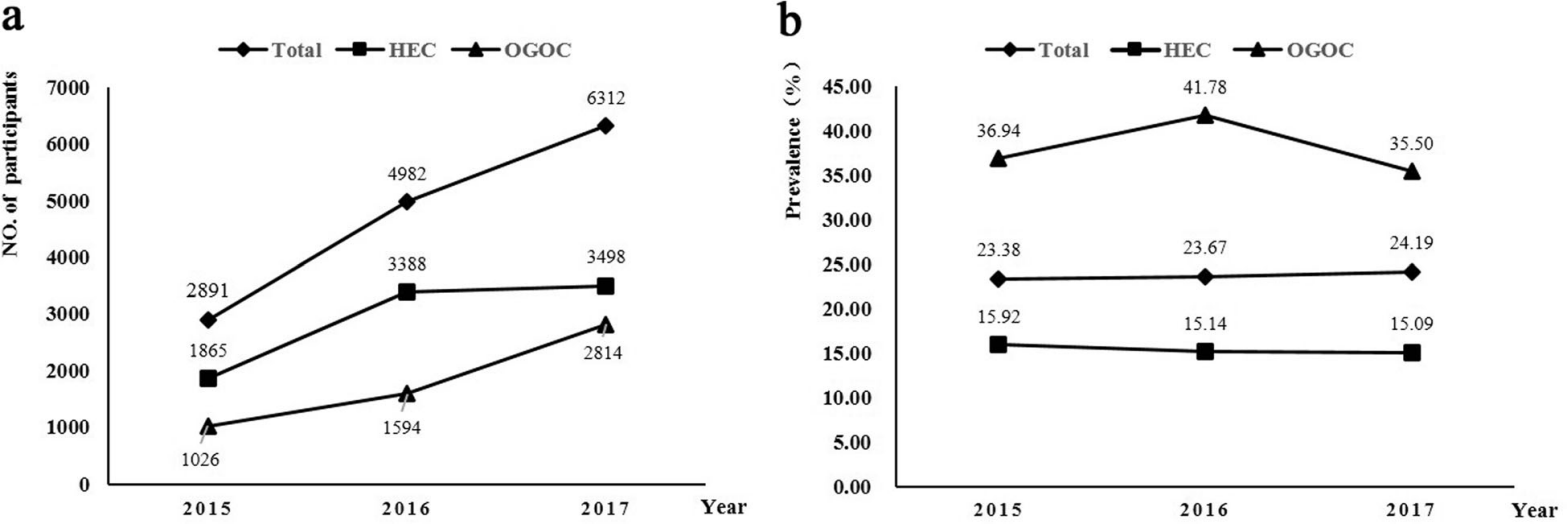
Prevalence of HPV infection change by year. (**a**: Number of women participate HPV genotyping test by year, **b**: Prevalence of HPV by year)

In order to inspect the infection of HPV in the past 3 years, we analyzed the data by years. As shown in Fig. 2, there was a mild uptick in the number of people who received HPV genotyping test, especially in the OGOC group. Although the HPV infection rate of OGOC group reached a peak in 2016, there was no significant change of that for total population during 2015 to 2017.

## Discussion

The HPV infection prevalence varies considerably from geographies to populations. Globally, the highest HPV infection rate was in Sub-Saharan (25.6%), followed by South America (14.3%), Asia (8.7%), and Europe (5.2%) [6]. Even within one country, the HPV infection rate varied. It was 28.4% in northeast China, 20.16% in south China and 14.2% in northwest China [15–17]. The prevalence of neighboring southwest provinces was26.2% in Chonqing, 16.95% in Guizhou, 12.9% in Yunnan [18–20]. Sichuan was the fourth largest population province located in southwestern China. Several studies have investigated the HPV epidemiology. However the results varied, the prevalence rate ranged from 10.15 to 31.3% [7–12]. Despite these studies, to the best of our knowledge, there was no previous study arising from large samples collected over a long period of time on both low risk and high risk HPV genotype distribution among both women that attended OGOC and HEC. In order to provide a more scientific basis for the prevention and control strategies of cervical cancer in Sichuan area, we performed this analysis. In our study, the HPV infection prevalence of the total population was 23.84%, which was basically consistent with the result of Li et al. (24.1%) [12]. After further analyzing, we found HPV infection prevalence in HEC group (15.29%) was significantly lower than that in OGOC group (37.62%). The prevalence of HPV infection of HEC population in our study was higher than that of He et al.’s research (12.6%) [9], which might due to that He et al.’s research calculated the high-risk HPV infection of OGOC population only. For OGOC population, our prevalence rate was slightly higher than that of Zhu et al.’s study (32.0%) [11]. Compared to other province of China, our HPV infection prevalence of HEC group was lower than that in Guangdong (17.25%) and Zhejiang (19.5%) [15, 21]. Our HPV infection prevalence of OGOC group was higher than that in Hubei (17.68%), Guangdong (20.16%) and Zhejiang (22.3%), but lower than that of Fujian (38.3%) [15, 22–24]. These might mainly be attributed to geographical variation. Thus, according to our data, women in Sichuan area were facing the great threat of HPV infection, especially the women undergoing gynecological clinical symptoms.

Considering the age of infected women, we found prevalence of HPV infection reached a peak at 21–30 years and 41–50 years group. This double-peak result was also found in other area of China, such as Hangzhou and Chongqing [20, 23]. However, after dividing the population into HEC or OGOC groups, we found the prevalence of HPV reached peak at 41–50 years group in HEC group, but at 21–30 years group in OGOC group. A possible explanation was young women were more sexually active. For example, they might have higher frequency of sex and more different sex partners. Besides, their immune systems were not sensitive to HPV infection, which resulted in the high infection rate in 21–30 years group. The high infection rate of 41–50 years group might due to reduced immune functions [23, 25, 26]. According to our data, women of 21–30 or 41–50 years old should pay more attention to HPV infection.

In our study, among the HPV positive population, we demonstrated that single genotype infection was the most, which were basically consistent with data of Tao et al. [27].

According to HPV prevalence survey, the most prevalent genotype worldwide was HPV 16 (2.41%), followed by HPV 58 (1.25%) and 31(1.07%) [6]. However several studies have demonstrated that HPV16, 52, 58 were the major infection genotypes in China [15, 22–24]. In our study, the top three prevalent HPV genotypes were HPV 52 (5.02%), 58 (3.61%), and 16 (3.24%) in the total screening population. Among the HPV positive women, HPV 52 was the most prevalent genotype in Sichuan area, which was consist with the data of Li et al. [12]. And this pattern was also showed in Yunnan and Guizhou Province. However, in a previous study by Sichuan, HPV 16 was identified to be the top prevalent genotype (19.4%) [27]. This might due to the bias of sample size and different survey period. Even though there were some discrepant results, the most prevalent genotype in Sichuan area were HPV 58, 16, 52. Among the low-risk HPV genotypes, HPV CP8304 was the most prevalent genotype in both HEC and OGOC group, which was consistent with Le et al.’s research [15]. In OGOC group, HPV 16, 18, 6, 11 genotype prevalence were significantly higher than in HEC group, while HPV 53 and CP8304 genotype prevalence were significantly lower than in HEC group. A possible explanation for the different prevalence of HPV 6 and HPV 11 in HEC and OGOC group might be that the infection of these genotypes were more likely to cause genital warts and made patients went to hospital for treatment. However, the reasons for these differences HPV prevalence between OGOG and HEC group remain unknown and should be investigated. In general, this information was meaningful for generating strategies for both cervical cancer screening and vaccine exploitation.

Persistent infection of high-risk HPV was a strong carcinogenic factor to induce cervical cancer. In present study, the prevalence of high-risk HPV was 81.41, 88.57, and 100% in ASCUS, LSIL and HSIL, respectively. The top genotypes were HPV52 in ASCUS, HPV 66 in LSIL, and HPV 58 in HSIL. HPV 52, 58, 16 accounted for 41.84% of cytology abnormalities. These results were consistent with the data of He et al., except for the HPV 66, 39 in LSIL, which may due to the bias of sample size [9].

Among histological abnormalities, the prevalence of high-risk HPV were 89.83, 96.30, 100, and 100% in CIN I, CINII, CIN III and CA. The top three genotypes were HPV 58 (21.62%), 52(27.03%), and 16(21.62%) in CIN I group, HPV 16 (70.59%), 52/33(23.52,23.52%), 51/39(11.76,11.76%) in CINIIgroup, HPV 58/16 (50,50%) in CIN III and HPV16/18/51/58 (25.00,25.00,25.00,25.00%) in CA group. HPV 52, 58, 16 accounted for 56.52% of histological abnormalities, which was consistent with several studies that investigated the relationship between HPV genotype and histological abnormalities in Sichuan area. These studies demonstrated that HPV 16, 58, 52 infection accounted for a large percentage of histological abnormalities [10, 11, 27–29].

HPV vaccine was introduced to China in 2017. The bivalent vaccine (Cervarix, targeted at HPV16/18) was available since August 2017, and the quadrivalent vaccine (Gardasil, targeted at HPV6/11/16/18) was available since December 2017 in Sichuan province [12]. Thus, our study was a survey before the mass use of HPV vaccine in Sichuan area. According to our study, these two kinds of vaccine cannot provide enough protection because of the high prevalent of HPV 52, 58 genotypes. Therefore, the nine-valent vaccine (Gardasil, targeted at HPV6/11/16/18/31/33/45/52/58) was more suitable for women of Sichuan province, which should be introduced as soon as possible. In addition, according to He et al.’s study, at least half women in western China were willing to take the HPV vaccine, because it would be beneficial to the prevention and treatment of HPV infection [30].

There were several limitations in this study. One was the absence of data from other areas in Sichuan province, which meant these results might not represent all women in Sichuan. Second, detailed information of population (e.g. number of sexual partners, smoking habits, etc.) was not available for us to evaluate the effect of these characteristics on the prevalence of HPV infection. Third, we have not performed the analysis on the genotypes distribution of per age group, which limited the understanding on the prevalence of HPV at every age stage. Last, the case of cervical cytology or histology abnormalities was limited. Therefore, larger and prospective studies about the mechanism are needed to validate our findings.

## Conclusions

Women in Sichuan area are facing great threat of HPV infection, especially the women undergoing gynecological clinical symptoms. Women with ages of 21 ~ 30 or 41 ~ 50 years old should pay more attention to HPV infection. The most prevalent genotypes in HEC group are HPV 52/CP8303/58, while in OGOC group are HPV 52/58/16. HPV 52/16/58 accounte for the majority of cytology and histological abnormalities. These data could provide a scientific basis for the prevention and control strategies of cervical cancer in Sichuan area.

## Supplementary information

**Additional file 1: Supplementary Figure 1.** Prevalence of HPV grouped by age in study population.

## Data Availability

The datasets used and analyzed in this study are available from the corresponding author upon reasonable request.

## Abbreviations

AGC: Atypical gland cells
ASCUS: Atypical squamous cells of undetermined significance
CI: Confidence intervals
CIN: Cervical intraepithelial neoplasia
DNA: DeoxyriboNucleic Acid
HEC: Health Examination Center
HPV: Human papillomavirus
HR-HPV: High-risk HPV
HSIL: High-grade squamous intra epithelial lesion
ICC: Invasive cervical cancer
LR-HPV: Low-risk HPV
LSIL: Low-grade squamous intraepithelial lesion
NILM: Negative of intraepithelial lesion or malignancy
OGOC: Obstetrics and gynecology outpatient clinics
PCR: Polymerase chain reaction
TCT: Thinprep cytologic test
