# Supplementary figures and images for: Prevalence and genotype distribution of HPV and cervical pathological results in Sichuan Province, China: a three years surveys prior to mass HPV vaccination
Source: Virol J. 2020 Jul 10;17:100. doi: 10.1186/s12985-020-01366-2 (PMC7350733; doi:10.1186/s12985-020-01366-2)

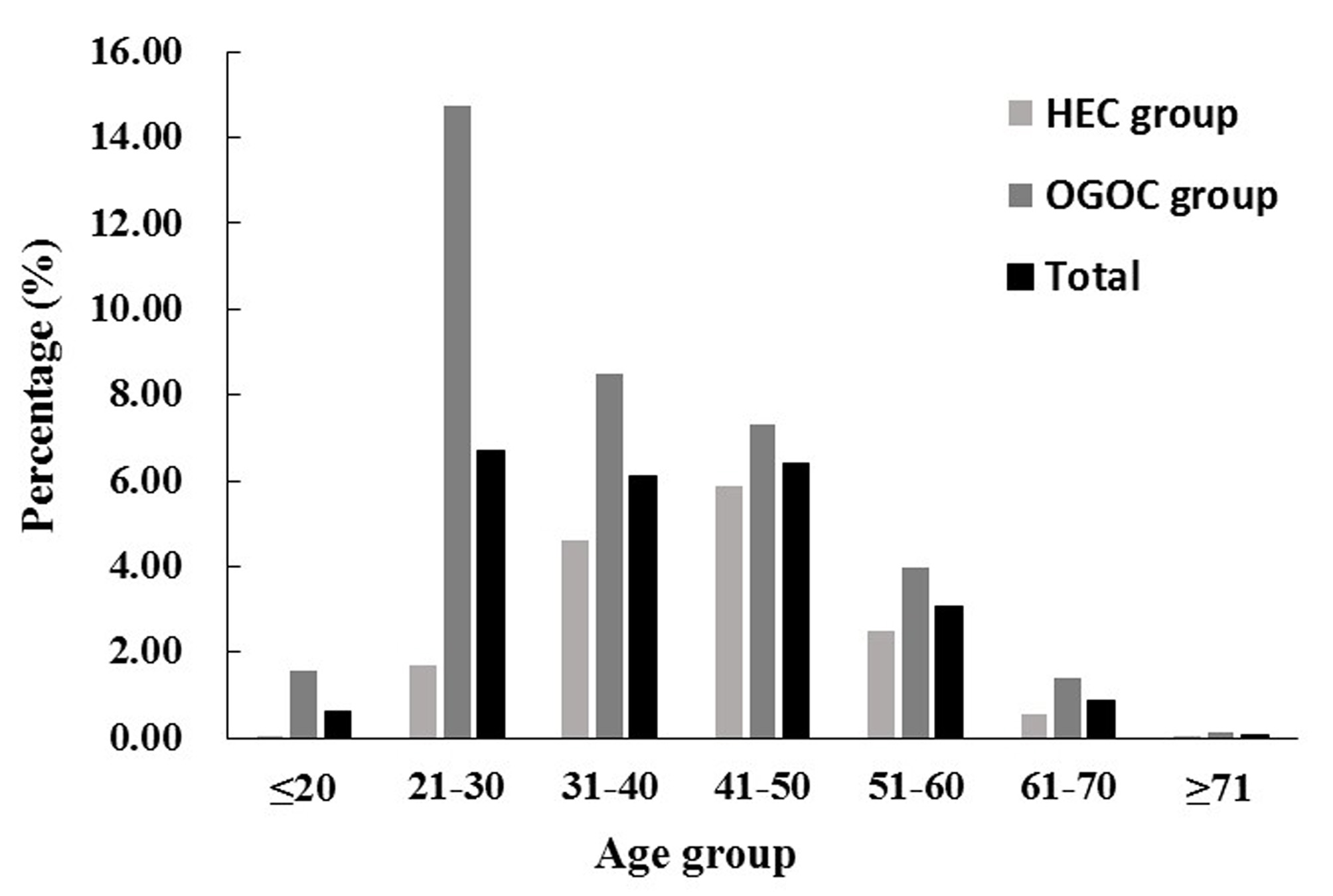

Supplement: Supplementary file 1 — Additional file 1: Supplementary Figure 1. Prevalence of HPV grouped by age in study population. [file 12985_2020_1366_MOESM1_ESM.tif]
